# How does government environmental investment promote green development: Evidence from China

**DOI:** 10.1371/journal.pone.0292223

**Published:** 2023-10-12

**Authors:** Qin Wang, Chao Zhou

**Affiliations:** Department of Graduate School, Sichuan International Studies University, Chongqing, China; Bucharest University of Economic Studies: Academia de Studii Economice din Bucuresti, ROMANIA

## Abstract

The *14th Five-Year Plan* stated that China should accelerate green development and promote a comprehensive green transition in economic and social development. As China’s economic growth slows, green development becomes greatly significant for the high-quality development of the economy. Based on China’s provincial panel data from 2005 to 2021, this study applies fixed effects model and mediating effect model to explore the influence of the government environmental investment on green development. The results indicate that (1) the government environmental investment was conducive to green development, but such effect weakened with time. (2) The government environmental investment indirectly promoted green development through the application of green patents and economic agglomeration. (3) The promotional effect of the government environmental investment varied according to region and time. Specifically, investment exerted the most significant effect on the green development of eastern China, which became more evident after 2015. The government should promote green development by implementing long-term assessment and accountability mechanisms, expanding the scale of economic agglomeration, improving the efficiency of the application of green patents, and improving the accuracy of environmental investment.

## Introduction

A favorable ecological environment is not only a vital prerequisite for human society to achieve sustainable development but also a realistic demand for people at a certain level of economic development. The factor-driven extensive growth model, which operates at the expense of the environment, is no longer suitable for China’s development in the new era. Therefore, the *14th Five-Year Plan* identified that accelerating green and low-carbon development and promoting the overall green transition of economic and social development is necessary. However, green development is systematic and complex, requiring the government’s active involvement in macro control. Environmental investment is an essential way for local governments to implement green development. It provides a solid material financial guarantee for green development and demonstrates the dual functions of promoting the transformation of industrial structure and regulating environmental resources. Although financial departments have been devoted to environmental protection in recent years, the situation remains severe. According to 2022 data from the Ministry of Finance (Source: Ministry of Finance, PRC, http://bgt.mof.gov.cn/zhuantilanmu/rdwyh/czyw/202301/t20230120_3863893.htm; Ministry of Natural Resources, PRC, https://www.mee.gov.cn/hjzl/sthjzk/sthjtjnb/.), the national general public budget expenditure exceeded 26 trillion yuan, of which environmental protection expenditure amounted to 539.6 billion yuan, while China’s ecological environment in terms of soil, water, and ecosystems still needs improvement. Therefore, further clarification of the relationship between the government environmental investment (GEI) and green development as well as the acting mechanism is needed in the current context. In summary, exploring strategies to better promote positive interactions between the two under increasingly prominent environmental pressures has become a crucial issue that urgently requires a clear response from relevant academic research, which is of great significance for promoting China’s high-quality economic development.

Therefore, this study takes 30 provinces in China as the research objective and utilizes their data from to 2005–2021 to explore the relationship between the GEI and green development as well as the mediating role of the green patent applications and economic agglomeration. It also investigates the effects of regional and temporal heterogeneity. Reasons for choosing provinces of China as the research objective may lie in two aspects. Firstly, given the vast territory of China, the overall study of GEI is more representative and comprehensive. Study only limited to certain area may be overly concrete and lacks applicability. Secondly, the environmental protection pressure of China remains heavy and the government environmental investment keeps growing. China’s economic growth has historically come at the expense of the environment, which has resulted in significant pollution and issues. Therefore, it is so vital to safeguard the environment that governments have made significant financial investments to support high-quality economic development while protecting environment. By doing so, this study aims to uncover the role of GEI, to further clarify its impact mechanism on green development and provide a theoretical basis and empirical support for the Chinese government to better understand the promotional effect of financial investment on green development and narrow the green development gap between targeted regions.

The main contributions of this study are as follows: First, it focuses on the economic effects of the GEI and its relationship with green development, which helps to supplement the literature on GEI as well as its impact and efficiency. Furthermore, this study analyzes the impact channels of GEI on green development from the perspectives of the application of green patents and economic agglomeration. This is conducive to a more comprehensive understanding of the relationship between government environmental investment and green development. It also interprets this impact mechanism from both theoretical and empirical perspectives, compensating for deficiencies in the existing literature to a certain extent. Finally, this study clarifies the existence of regional heterogeneity in the role of the GEI in promoting green development. Based on this, strategies to promote green development are proposed, which are helpful in providing relevant policy references for narrowing regional differences and alleviating the imbalance of regional green development. As the Chinese government is paying increasing attention to the ecological environment, giving full play to the positive role of the GEI in promoting green development and enhancing the positive interaction between them in the targeted regions will further improve the high-quality development of China’s economy.

## Literature review

Domestic and foreign scholars have conducted many studies on GEI and green development, which are summarized as follows:

The first category concerns green development and primarily focuses on its influencing factors and the government’s policy-guiding role. Initially, scholars focus on the Chinese government’s policy guidance in promoting green economic development. Green financial policies such as green credit can leverage private capital into green development, which broadens the financing channels of enterprises, enhances their green productivity, and reduces carbon emissions, thus promoting the green transformation of the economy [1–3]. Moreover, scholars have mainly analyzed the impact of the digital economy, technological innovation, and environmental regulation on green development. Through studies on China, India, and OECD member countries, Grover, Mensah et al., and Wei and Hou concluded that the digital economy and technological innovation promote green development, while there is heterogeneity and a time lag in the former [4–6]. However, researchers hold various views on environmental regulations. Environmental regulation is argued to inhibit regional green development because it increases firms’ production costs, thus weakening their market competitiveness [7]. Environmental regulations also have adverse spatial effects, as environmental regulations in neighboring areas hinder local green development and exert a more significant negative spatial spillover effect in economically neighboring areas than in geographically neighboring areas [8]. However, other scholars believe that the relationship between environmental regulations and green development is nonlinear. Overly harsh environmental regulations hinder urban green development, while appropriate environmental regulations indirectly promote regional green transformation through spillover demonstration, factor substitution, and technological innovation effects [9, 10].

The second category is the study of the GEI, which is generally considered to be environmentally effective and affected by several factors. First, GEI is conducive to government enforcement restricting carbon emissions and reducing haze through technological innovation [11, 12]. Based on the positive effects of GEI, scholars have further studied its investment efficiency and influencing factors. The overall efficiency of GEI in China is low, and significant regional differences caused by technical efficiency exist [13]. During this process, the GEI is affected by several factors. External factors include foreign direct investment, urbanization level, and digital economy development, whereas government intervention is the internal factor [14–16].

Finally, research on the relationship between government environmental investment and green development is the third category. Few in-depth analyses are available on this topic because current studies mainly concentrate on the relationship between the government’s other economic actions and green development. GEI significantly impacts the green economy. Increasing the proportion of fiscal environmental protection expenditures to total fiscal expenditures positively contributes to green technological progress and economic growth [17]. Whether or not the expenditure is productive, fiscal competition among governments significantly enhances green development, and tax competition strengthens the effect of fiscal decentralization on green development [18, 19]. In this process, the economic behavior of local governments is largely determined by their economic expectations. Local governments’ economic expectations significantly inhibit the efficiency of green development because it leads local governments to excessively pursue rapid economic growth at the expense of green transformation [20].

The literature review indicates that domestic and foreign scholars have conducted much in-depth research on green development and GEI, providing valuable references for this study. However, gaps remain in the literature. First, existing studies have primarily focused on the environmental effects of the GEI, with insufficient attention paid to its economic effects. Second, existing studies concentrate on the relationship between environmental regulation and the government’s other economic behavior and green development but fail to deeply explore the relationship between green development and GEI, which is closely related to the environment. Moreover, the impact mechanism between the two requires further analysis. Based on this, China’s provincial panel data from to 2005–2021 was used in this study to analyze the relationship between GEI and green development, as well as its impact mechanism, to provide theoretical support to further strengthen the positive interaction between the two and boost the green transformation of China’s economy.

## Theoretical analysis and hypotheses

Regional green development focuses on resolving existing environmental pollution issues, developing and applying green technologies, and improving and constructing environmental infrastructure. Environmental pollution issues are mainly addressed as negative external effects through taxation or subsidies, internalization of external effects, and the determination of property rights [21]. This requires increased investments in environmental management and protection. Given that the ecological environment is a public good with a strong public interest in governance, private capital, such as enterprises, lacks the enthusiasm and initiative to invest. Finance is an efficient tool for government macro-control because it can compensate for the private sector’s underinvestment and effectively repair the negative external impacts of economic activity on the ecological environment brought on by the “tragedy of the commons.” In addition, the GEI can successfully promote the R&D of diverse green technologies, fundamentally reinventing outdated manufacturing techniques, while providing direct financial support for environmental management. Green technological innovation is an indispensable foundation for green development. However, as a highly innovative activity, green technological innovation has significant capital demand, a long benefit recovery period, intense uncertainty, and a weak willingness of social capital to invest. In this situation, government investment is required to ease critical organizations’ funding shortages and enhance financial support for the application of green technology [22]. However, the promotion and use of green technology innovation strongly emphasizes sensible resource management and stringent limits for pollutant emission levels. Therefore, considering possible market failure, the “promising government” must strengthen support for green financial expenditures such as environmental investment to stabilize the expectations of relevant market economic entities and ensure that the “effective market” plays a better role, to promote the green development. Finally, with a significant guiding effect, government investment drives more social investment to satisfy project financing needs in infrastructure, public services, and industry. By making significant environmental investments, local governments assist in greening businesses, developing environmental infrastructure, and mobilizing additional social capital, particularly private capital, for environmental protection and green development. Therefore, the first hypothesis is proposed.

Hypothesis 1: GEI promotes green development.

Green technology is a key factor in China’s establishment of an ecological civilization and high-quality green development. An essential strategy for driving green technology is to promote and utilize innovative green patents that are oriented towards environmental protection. However, their practical applications involve a complicated and ongoing commercialization process from product R&D to pilot testing to industrialization that spans a significant amount of time. Financial investment must be increased, particularly in the pilot stage, to fully offset capital losses in crucial links. In addition, traditional financial institutions and private capital show less interest in investment because of the high level of uncertainty concerning the application of green patents. In this situation, government finance, as an essential tool for adjusting investment structures, plays a more significant political role than economical, primarily by fostering an environment in which the application of green patents is promoted [23]. The government specifically boosts its support for and focuses on the application of green patents by establishing special funds, including green banks and green funds. A favorable atmosphere for their application is produced via pertinent policies on platforms and abilities. Additionally, relevant businesses actively invest under the influence of “policy incentives,” creating a diversified multi-level financial guarantee framework for application. Thus, the second hypothesis is proposed.

Hypothesis 2: GEI promotes green development through the application of green patents.

This study explains three aspects of the effect of economic agglomeration on the relationship between the GEI and green development: competition, scale, and innovation effects. First, the competitive effect is analyzed. Economic agglomeration can encourage the concentration of local businesses and industries in particular areas, which causes rivalry among different producers in terms of price and quality. This competitive effect can constitute an efficient energy-saving incentive mechanism, driving businesses to update energy-intensive technologies and equipment impulsively in response to rising traditional energy prices and more stringent environmental laws [24]. In addition, an analysis of the economies of scale is presented. Energy production and environmental management units can exchange raw materials and production factors, such as production machinery, labor, and technology, on a larger scale in economic agglomeration zones, thereby lowering the production energy consumption per unit of product. In addition, economically concentrated areas have a more diversified system of division of labor and relatively superior environmental infrastructure, which can further exert scale effects [25]. The government may encourage a large number of businesses to relocate through advantageous policies for companies in such regions, such as green project subsidies and tax credits. As a result, the assembled enterprises need less time and space owing to their geographical proximity and share the economic benefits brought about by the green transformation of surrounding enterprises. Finally, the innovation effect is analyzed. The GEI prioritizes the development of high-quality talent in green technology discovery and commercialization, in addition to providing financial support for businesses’ transition to a greener business model. Economic agglomeration can encourage the movement of human capital into the agglomeration area, and the influx of top personnel can offer advanced technology and management experience needed for a green revolution. Technological advancements made by businesses in the ecological and environmental fields are easily absorbed and swiftly spread to neighboring businesses, promoting the green transformation and growth of the entire region through imitation learning. Therefore, this paper proposes the following third hypothesis:

Hypothesis 3: GEI promotes green development through economic agglomeration.

## Research design

### Models

#### Benchmark model

The following benchmark model was created to examine the first hypothesis about the impact of the GEI on green development.

$$gtfp_{it}=c+\beta_{1}rein_{it}+\beta_{2}ind_{it}+\beta_{3}urb_{it}+\beta_{4}\;\text{ln}\;pgdp_{it}+\beta_{5}\;\text{ln}\;rd_{it}+\varepsilon_{it}$$ (1)

Here, *i* and *t* represent the time and provinces of all samples. *gtfp* is the green development level, the explained variable of this paper. *rein* represents the GEI, the explanatory variable of this paper. Control variables that may influence the green development are included. *ind* reflects the industry proportion of each province. *urb* is the urbanization level of these provinces. *Pgdp* and *rd* represent the economic development level and R&D investment of companies of each province. *Ε* is the random error term. *Β*_1_ demonstrates the effect of government environmental investment on green development. If *β*_1_ remains greater than 0 when all control variables are unchanged, GEI is conducive to green development. If not, such investment does not affect green growth.

#### Mediating effect model

The theoretical analysis demonstrates how the GEI encourages green development through the application of green patents and economic agglomeration. A mediating effect model was used to test the second and third hypotheses. The formulae are as follows:

$$gtfp_{it}=\alpha_{0}+\alpha_{1}rein_{it}+\beta_{1}ind_{it}+\beta_{2}urb_{it}+\beta_{3}\;\text{ln}\;pgdp_{it}+\beta_{4}rd_{it}+\mu_{it}$$ (2)

$$mediator_{it}=\alpha_{0}+b_{1}rein_{it}+\beta_{1}ind_{it}+\beta_{2}urb_{it}+\beta_{3}\;\text{ln}\;pgdp_{it}+\beta_{4}rd_{it}+\varepsilon_{it}$$ (3)

$$gtfp_{it}=\alpha_{0}+c^{\prime }rein_{it}+c_{1}mediator_{it}+\beta_{1}{\text{ind}}_{it}+\beta_{2}urb_{it}+\beta_{3}\;\text{ln}\;pgdp_{it}+\beta_{4}rd_{it}+\varpi_{it}$$ (4)

Here, *mediator* represents two variables including the application of green patents (*pau*) and economic agglomeration (*ene*). Other variables are the same as in Eq (1). Eq 2) is estimated first. Only when *α*_1_ is significantly positive would the Eqs (3) and (4) be tested. If *b*_1_ and *c*_1_ are significant, GEI plays a positive role in green development through the application of green patent and economic agglomeration. *C*′ in Eq (4) indicates whether the mediating effect is full or partial mediation.

### Variable specification

The explained variable was the green development level (*gtfp*). Referring to Li [26] and Li and Li [27], the green total factor productivity is used to measure the green development level of a region. In contrast to traditional total factor productivity, green total factor productivity adds pollution emissions to the measurement system, allowing for a more thorough depiction of a nation’s or region’s industrial and economic progress. The non-energy inputs in this study include capital and labor inputs, which are expressed by the labor force and capital stock in each province. Capital stock data were calculated using the perpetual inventory method of Zhang et al. [28], assuming a capital depreciation rate of 9.6%; all data were converted to 2000 price levels. The expected output of this study, the real gross product (100 million yuan) of each province, was converted to 2000 price levels. Owing to the availability of data, this study used carbon emissions (million tons), industrial sulfur dioxide emissions (10 thousand tons), wastewater emissions (10 thousand tons), and general industrial solid waste emissions (million tons) of each province to measure the undesired output.

The explanatory variable in this study is the government environmental investment (*rein*). To improve comparability and eliminate differences caused by various population, this paper measured GEI by the ratio of government environmental investment to the population with reference to Cao et al. [29].

Control variables are as follows. Referring to previous research [30–32], this study selected the following indicators as control variables to alleviate the differences among provinces. (1) Industry proportion (*ind*). The higher the industry share in the economy, the stronger is the dependence on traditional energy sources, which, in turn, affects green development. This was calculated using the ratio of industrial added value to GDP. (2) Level of urbanization (*urb*). Urbanization facilitates factor mobility and resource allocation, which influence green development. This was calculated using the urbanization rate. (3) Economic development (*pgdp*). The level of regional economic development is closely related to green development; however, simple economic growth at the cost of energy consumption may not fully reflect green economic growth. Therefore, it was evaluated by the per capita gross domestic product and presented in logarithmic form. (4) R&D investments (*rd*). Technological progress helps enterprises improve their technological levels and energy efficiency, thereby reducing pollutant emissions. This is measured as the ratio of internal R&D expenditure to GDP in industrial enterprises and presented in logarithmic form.

Mediators are listed below. According to prior investigations, the application of green patents and economic agglomeration are two ways in which the GEI may contribute to green development. Consequently, in this study, two indicators were chosen as mediators. (1) Application of green patents (*pau*). Considering that this study mainly examines the mediating effect of green patent applications, it uses the number of authorized green patents per 10,000 people as a measurement, with reference to Zhang and Hu [33]. Green patents include green inventions and utility patents. (2) Economic agglomeration (*ene*). Restricted by the data, this study, referring to Zhang [34] and Chen [35] who measured economic agglomeration based on urban-rural disparities, employed the ratio of urban residents’ disposable income to rural residents’ disposable income as a measurement.

### Data specification

Based on the *China Statistical Yearbook*, *China Environment Statistical Yearbook* and the *China Energy Statistical Yearbook*, the authors obtained the required data for 31 provinces in China (excluding Hong Kong, Macao, and Taiwan). However, Tibet was excluded owing to missing data. Therefore, the sample data for this study include 30 provinces in China from to 2005–2021. In addition, the data was subdivided into eastern, central, and western regions (Eastern regions: Beijing, Fujian, Guangdong, Shanghai, Jiangsu, Zhejiang, Liaoning, Hainan, Tianjin, Hebei, Shandong. Central regions: Anhui, Henan, Heilongjiang, Hubei, Hunan, Jilin, Jiangxi, Shanxi. Western regions: Chongqing, Yunnan, Xinjiang, Sichuan, Shaanxi, Guizhou, Qinghai, Ningxia, Inner Mongolia, Gansu, Guangxi.). In order to reduce the effect of spurious outliers, this article winsorized all variables at 1%. The descriptive statistics of each variable are shown in Table 1.

**Table 1:** Results of descriptive statistics.

Variable	Description	Observed Value	Mean	Standard Deviation	Minimum	Maximum
**gtfp**	Green development level	510	1.444	0.467	1	3.628
**rein**	GEI	510	5.613	4.814	0.537	26.122
**ind**	Industry proportion	510	35.489	8.539	11.838	54.227
**urb**	Level of urbanization	510	55.388	13.860	29.884	89.309
**lnpgdp**	Economic development	510	10.500	0.661	8.996	11.961
**lnrd**	R&D investment	510	4.430	0.599	2.811	5.560
**iein**	GEI of industrial added value	510	6.959	8.005	0.215	47.016
**ene**	Economic agglomeration	510	2.703	0.460	1.855	4.114
**pau**	Application of green patents	510	0.775	1.188	0.0156	6.437

## Results and discussion

The empirical test was conducted in three steps. First, the hypothesis that GEI encourages green development was examined. The second step was to gauge the reliability of this estimation. The purpose of the final section was to test the main thesis, which states that GEI promotes green development through the application of green patent and economic agglomeration. This study adopted a robust standard error in the regression analysis and conducted autocorrelation testing for the variables to address potential heteroscedasticity. The autocorrelation test results showed that there was a correlation between variables, but no significant multicollinearity because the VIF value was 3.56 (This paper does not report this result due to space constraints. Information is available for readers upon request.).

### Benchmark model regression

Panel regression was employed to analyze data from 30 Chinese provinces from 2005 to 2021 to determine whether the GEI could promote green development. GEI is the explanatory variable, and the level of green development is the explained variable. Two estimation techniques, random effects (Re) and fixed effects (Fe), were applied based on regression model (1). Each technique was estimated with and without control variables. The *p* value was zero for the Hausman test, indicating that the initial hypothesis should be rejected. A fixed effects model is recommended. Table 2 presents the specific outcomes. Columns (3) and (4) show that the coefficients on *rein* are 0.032 and 0.012, respectively. When control variables such as industrial proportion, urbanization level, economic development level, and human capital level are included, the coefficient of the GEI is significantly positive at the 1% level. These findings show that increasing government environmental spending supports regional green development, thus confirming hypothesis 1. This conclusion is contrary to that of Ye and Guo [17], who used data from to 2006–2017 to analyze the relationship between total fiscal environmental expenditure and green growth. The possible reason is that there is a particular “crowding out effect” of fiscal environmental expenditure in the earlier years, which is detrimental to the increase in corporate environmental investment, thus preventing green technological progress and green total factor productivity. However, the efficiency, investment structure, and regulation of the GEI have improved after 2017 due to better implementation of the concept of green development [36], thus helping green economic growth.

**Table 2:** Results of benchmark model.

Variables	(1)RE	(2)RE	(3)FE	(4)FE
**rein**	0.032***	0.012***	0.032***	0.012***
	(8.57)	(3.21)	(8.57)	(3.21)
**ind**		-0.009***		-0.009***
		(-3.70)		(-3.70)
**urb**		0.012***		0.012***
		(5.07)		(5.07)
**lnpgdp**		0.028		0.028
		(0.40)		(0.40)
**lnrd**		0.089**		0.089**
		(2.10)		(2.10)
**Constant**	1.164***	0.397	1.266***	0.382
	(17.96)	(0.72)	(48.72)	(0.64)
**Observations**	510	510	510	510
**R-squared**			0.130	0.351

Note:

***, **, and * indicate significance at the 1%, 5%, and 10% levels, respectively. The numbers in parentheses are T values. Below is the same.

Regarding the control variables, the coefficient of urbanization was 0.012 and significantly positive at the 1% level. This finding implies that urbanization encourages green development. This might be because it promotes the rapid flow of production factors, such as labor and capital. It may also enhance the green transformation of local businesses and industries, while transforming old industries and enhancing and eradicating backward production capacity. In addition, the coefficient of industry proportion was significantly negative, indicating that it does not favor the advancement of green development. In other words, the more industrial added value there is in the local economy, the more local green development is hindered. Additionally, the coefficient of R&D investment was significantly positive at the 5% level, indicating that it promotes green development. Finally, although the coefficient of industrial percentage is positive, it does not pass the significance test, meaning that it does not favor the advancement of green development. This may be because China’s economy develops as energy consumption increases, and high energy-consuming and carbon-emitting activities expand, thus hindering green development in the short term.

### Robustness test

#### Lag effect

The GEI could offer financial assistance for green development in environmental pollution control, green enterprise transformation and the application of new energy, and resolve the issue of insufficient funding in the early stages of regional green development. Capital investment, however, has an inertial delay characteristic, which suggests that previous government environmental expenditures may theoretically affect current green development. To test the findings, this study incorporated the current period and lag period I and employed fixed and random effects models. According to the Hausman test results, the *p* value is 0. This makes use of a fixed effects model. The regression results are displayed in Column (1) of Table 3.

**Table 3:** Robustness test.

Variables	(1) Lag Effect (FE)	(2) Lag Effect (RE)	(3) Variable Changing	(4) GMM
**rein**	0.013***	0.013***		0.012*
	(3.61)	(3.61)		(1.79)
**L.rein**	-0.009**	-0.009**		
	(-2.56)	(-2.56)		
**ind**	-0.009***	-0.009***	-0.006**	-0.009***
	(-3.37)	(-3.37)	(-2.31)	(-3.53)
**urb**	0.010***	0.010***	0.015***	0.012***
	(4.40)	(4.40)	(6.68)	(5.27)
**lnpgdp**	0.047	0.047	-0.035	0.028
	(0.66)	(0.66)	(-0.50)	(0.43)
**lnrd**	0.087*	0.087*	0.020	0.089**
	(1.91)	(1.91)	(0.48)	(2.03)
**iein**			0.011***	
			(5.40)	
**Constant**	0.293	0.282	0.994*	0.397
	(0.49)	(0.50)	(1.66)	(0.78)
**Observations**	493	493	510	510
**R-squared**	0.360		0.374	0.588

As shown in Table 3, the coefficient of GEI is 0.013 and passes the 1% significance test, demonstrating that it positively impacts green development in the current period. In other words, the conclusions of this study are consistent. Notably, the lag period I coefficient is negatively significant and lower than that in the current period, indicating that the positive effect of GEI decreases with time. This finding differs from that of Ma and Burna [37]. One possible reason is that their research sample was limited to the Yangtze River Delta Urban Agglomeration, and the relationship between environmental protection expenditures and green development has geographical specificity. Across the entire country, this phenomenon may be caused by a variety of factors. First, the investment in the ecological environment is substantial and has a long cycle. The economy is experiencing an increasing downward pressure. Consequently, the local governments’ follow-up funding was erratic and inconsistent [38]. Additionally, although the government has invested significantly in environmental preservation, its implementation requires improvement. The long-term environmental accountability and assessment framework must also be strengthened and implemented. The empirical results presented above demonstrates that GEI favorably influences green growth through the application of green patents and economic agglomeration. Its limited long-term impact can be ascribed to the inhibition of its mechanism of action.

#### Change of explanatory variables

Referring to Zhang et al. [39], hypothesis (1) was re-examined with iein, the environmental investment of industrial added value. According to the findings presented in Column (3) of Table 3, the iein has a coefficient of 0.011, which is positive at the 1% level. This finding supports the hypothesis that the GEI encourages green development.

#### Treating endogeneity problems

Considering that missing variables and measurement errors cause endogeneity problems with inconsistent estimation results, instrumental variables were introduced to conduct a robustness test for potential endogeneity problems. Compared to estimation methods such as two-stage least squares, the biggest advantage of the generalized method of moments (GMM) is that it does not specifically restrict the overall distribution, focusing only on certain characteristics such as moment conditions, which offer the estimated value of a property of a large sample. Referring to Khan et al. [40] and Fan and Peng [41], this study uses the one-phase lag of the core explanatory variable as an instrumental variable. The specific outcomes of the GMM regression are displayed in column (4) of Table 3. It is clear that the GEI has a coefficient of 0.012, which is significantly positive at the level of 10%, further demonstrating the power of GEI to greatly advance green development.

### Mediating effect test

The panel regression results verified the first hypothesis. GEI plays a positive role in promoting green development; however, this effect is indirect. Therefore, the two approaches were investigated separately, based on the indirect impact mechanism of the GEI. Table 4 shows the precise estimation results.

**Table 4:** Results of mediating effect model.

Variables	(1) gtfp	(2) pau	(3) gtfp	(4) ene	(5) gtfp
**rein**	0.012***	0.025***	0.007**	0.011***	0.011***
	(3.21)	(2.87)	(2.21)	(2.88)	(2.92)
**ind**	-0.009***	-0.039***	-0.003	-0.010***	-0.008***
	(-3.70)	(-6.57)	(-1.06)	(-3.70)	(-3.31)
**urb**	0.012***	0.001	0.011***	-0.016***	0.013***
	(5.07)	(0.21)	(5.46)	(-6.62)	(5.48)
**lnpgdp**	0.028	1.098***	-0.162**	-0.142*	0.041
	(0.40)	(6.65)	(-2.44)	(-1.96)	(0.59)
**lnrd**	0.089**	0.423***	0.016	-0.048	0.093**
	(2.10)	(4.21)	(0.40)	(-1.08)	(2.21)
**pau**			0.173***		
			(9.92)		
**ene**					0.091**
					(2.10)
**Constant**	0.382	-11.426***	2.356***	5.564***	-0.123
	(0.64)	(-8.12)	(4.09)	(8.99)	(-0.19)
**Observations**	510	510	510	510	510
**R-squared**	0.351	0.491	0.460	0.423	0.356

The estimation results are shown in columns (1) to (3), with the application of green patents acting as the mediator; and in columns (1), (4), and (5), with economic agglomeration acting as the mediator. The regression results from the first phase were validated in a previous study following the mediating effect verification approach. To determine whether the GEI had a meaningful effect on the two mediators, the second step was to test Eq (3). Columns (2) and (4) show the results. For the application of green patents and economic agglomeration, the coefficient of the GEI is significantly positive at the 1% level, indicating that GEI can drive the application of green patents and improve the degree of local economic agglomeration. Finally, the third step was applied to the test. Variables including the GEI, green development, and mediators were included in the same regression formula. Columns (3) and (5) display the projected outcomes. The estimation coefficients for *pau* and *ene* are 0.173 and 0.091, respectively, and at least pass the 5% significance test. This finding suggests that economic agglomeration and the application of green patents can help advance regional green development. Additionally, for the two action routes, the GEI coefficients were significantly positive at the 5% level, indicating the existence of partial mediation. This analysis implies that the GEI is conducive to regional green development through the application of green patents and economic agglomeration. Thus, the second and third hypotheses are verified.

### Heterogeneity analysis

#### Regional heterogeneity

GEI plays various roles in green development with different effect intensities because economic development, the level of government investment, and tax policies differ from region to region. Therefore, this study further separated the data into eastern, central, and western regions. The regression results are presented in Table 5.

**Table 5:** Regional heterogeneity.

Variables	(1) Eastern Part	(2) Central Part	(3) Western Part
**rein**	0.040***	0.002	-0.002
	(3.67)	(0.15)	(-0.44)
**ind**	0.006	-0.007*	-0.000
	(0.49)	(-1.68)	(-0.07)
**urb**	0.016**	0.017***	0.004
	(2.29)	(3.68)	(0.60)
**lnpgdp**	0.162	0.887***	-0.004
	(0.74)	(3.68)	(-0.02)
**lnrd**	-0.099	-0.314**	0.064
	(-0.54)	(-2.28)	(1.01)
**Constant**	-1.142	-7.010***	0.948
	(-0.58)	(-3.42)	(0.67)
**Observations**	187	136	187
**R-squared**	0.274	0.234	0.034

The GEI coefficients in the central and western regions fail to pass the significance test, with coefficients of 0.002 and -0.002, respectively, indicating that GEI has not significantly impacted the central and western regions yet. Simultaneously, according to the sign of the coefficient, the correlation between GEI and green development in the western region is negative. This conclusion is similar to the one drawn by Li and Bai [42] when analyzing the relationship between industrial policy and green competitiveness of enterprises, that green development in the central and western regions needs to be improved. There are two possible reasons for this. Firstly, compared to the eastern region, the efficiency of environmental protection expenditure in the western and central regions was relatively low. Although the government has invested a large amount of funds, the utilization efficiency and accuracy require improvement. Secondly, government subsidies can easily lead to fraudulent subsidies received by enterprises and the use of subsidy funds for other high-income channels unrelated to emissions reduction, resulting in high costs but poor effectiveness of the GEI [43]. For the eastern region, the coefficient of the GEI has a 1% significance. This finding shows that such investments positively influence green development, with investment benefits more evident in eastern China. This is because the eastern region has more economic and geographical advantages, with higher levels of green finance and economic development than the central and western regions. Therefore, local governments pay more attention to the ecological environment, with more GEI and intense public awareness of environmental protection.

#### Temporal heterogeneity

At different times, the national emphasis on the ecological environment and the environmental investment behavior of local governments varies greatly, causing temporal heterogeneity in green development. In 2012, the 18th National Congress of the Communist Party of China included the construction of ecological civilization in the overall layout of the “Five in One” for the first time, elevating it to a national strategy. Concepts like “clear waters and green mountains are as good as mountains of gold and silver” became more popular than before. Besides, the *Integrated Reform Plan for Promoting Ecological Civilization* was issued in September 2015 to implement this spirit. At the same year, the concept of green development was also officially stated. Both of them proposed higher requirements for protecting the ecological environment in various regions. Therefore, this study divided the data into three periods for analysis, 2005–2011, 2012–2014, and 2015–2021. The results are summarized in Table 6.

**Table 6:** Temporal heterogeneity.

Variables	(1) 2005–2011	(2) 2012–2014	(3) 2015–2021
**rein**	0.002	-0.001	0.018**
	(0.30)	(-0.23)	(2.04)
**ind**	0.000	-0.010***	-0.016
	(0.08)	(-3.37)	(-1.60)
**urb**	0.011***	0.013***	0.025***
	(6.89)	(4.63)	(3.71)
**lnpgdp**	-0.098**	0.188**	0.230
	(-2.04)	(2.16)	(1.15)
**lnrd**	-0.010	0.027	0.114
	(-0.43)	(0.45)	(0.84)
**Constant**	1.772***	-1.051	-2.533
	(4.48)	(-1.37)	(-1.41)
**Observations**	210	90	210
**R-squared**	0.501	0.777	0.332

During 2005–2011 and 2012–2014, the coefficient of GEI decreased, and its sign changed from positive to negative, indicating that although the 18th National Congress of the Communist Party of China clearly proposed to vigorously promote the ecological civilization construction, local governments did not perform well in protecting ecological environment. Supported by the research of Wu and Qian, this finding might be explained by the shortage of regulations and proper management over such a short period [44]. Fortunately, this was achieved in the third stage. The year 2015 was a watershed for regional green development. The coefficient of the GEI is positive and significant at the 5% level. The results indicate that the concept of green development and the strict *Reform Plan* forced local governments to focus on regional environmental protection and invest in green transformation and development, thus playing a positive role in green development in the region.

## Conclusion and implications

### Conclusion

As an environmental-friendly and sustainable way of development, green development exerts an important impact on achieving the strategic goal of “carbon peak and carbon neutrality” and promoting high-quality economic development. This study tested the mechanism of the GEI in green development using the fixed effects model and a mediating effect model based on panel data of 30 Chinese provinces from 2005 to 2021. Regions and times were included in the heterogeneity test. The results of this study are as follows: First, green development is encouraged by government environmental investment. The findings are robust even after switching the measurement. Note that the positive effect of the GEI decreases over time and cannot support green development further. Second, the transmission mechanism demonstrates that the environmental investments made by the government would enhance the environment through the application of green patents and economic agglomeration. Finally, according to the heterogeneity analysis, green development in eastern China was more obviously affected by the GEI, and such investment played a more significant role in promoting green development after 2015. The following recommendations are made based on the research findings:

### Implications

Scaling up input and improving the level of administration. First, provinces, cities, and counties must develop ecological and environmental protection initiatives according to their administrative divisions and geographical settings. It is preferable to encourage regional businesses to participate in project governance. Governments at all levels should allocate specific funds for environmental protection and offer financial support through grants and subsidies, project subsidies, and tax breaks to ease the financial burden on businesses caused by environmental management. More favorable policies and funding must be provided to areas with major pollution problems and remarkable control successes to reduce regional ecological and environmental pollution, point by point and level by level. In addition, establishing a sound green performance assessment mechanism, rating evaluation mechanism, and long-term environmental accountability mechanism and regularly verifying the status and purposes of environmental protection funds is prudent. Priority support should be given to policies and projects with high performance levels, in principle, while those with low efficiency and high repetition should be cancelled. Conducting in-service and resignation reviews of the performance of relevant ecological and environmental leaders is also vital. The leaders of projects with unclear statuses and insignificant benefits should be interviewed. Those who fail to perform by themselves should impose administrative penalties.

Fully realizing the mediating effect of economic agglomeration and green patent application. Units at all levels must be urged to overcome administrative divisional barriers and share infrastructure like sewage treatment infrastructure to enable companies to congregate in one location. Companies with significant upstream and downstream correlations within an industry or considerable inter-industry contact should be placed compactly. Businesses in the cluster region should receive specific policy and financial support in domains such as the green project declaration, environmental tax exemptions and subsidies, and business finance. However, hastening the application for green patents and boost the effectiveness of low-carbon technological applications is preferable. First, scientific research institutes, colleges, universities, and businesses, should have access to platforms to innovate green technologies. Several units must encourage policy and financial support for patents, as well as the R&D of low-carbon technology. New-generation information technologies, such as blockchain and 5G, should be introduced to explore new application scenarios and provide convenience for specific applications of green technology. Second, patent owners and related companies should be urged to open innovative low-carbon patents to promote the broader adoption of green technology. The government should pay a patent transfer fee at least as high as the market rate or offer open business policy convenience to encourage the flow of patents into the market. Finally, the threshold for obtaining green invention patents should be raised. Patents with specific possibilities for future use should be produced and green patents that meet the criteria for timely application and ecological value should be awarded and supported.

Improving the accuracy of government environmental investment by adopting measures for local conditions. Significant disparities exist in terms of geography, economic development, and financial soundness between China’s eastern, central, and western regions. Consequently, the effect of GEI on green growth also varies. For the Eastern region, which has a robust economy and many environmental-friendly businesses, fully utilizing its skills and industrial advantages is important. Additionally, it should take the lead in promoting low-carbon technologies and environmental management experiences to help solve environmental issues in the central and western regions. Besides, a sound policy for green talents concerning their living, education and family should be established to attract more enterprises and high-quality labor. Establishing strict environmental standards concerning aspects such as sewage treatment and carbon emissions for heavy-industry companies is also necessary. In addition, financial support from green banks and funds should be provided to assist these businesses in updating their production machinery and developing new production techniques. Finally, the government should encourage firms in eastern China to collaborate with other companies to form a support system.

In summary, this article discussed the relationship and mechanism concerning the GEI and green development and empirically tested relevant hypotheses. This study established a preliminary theoretical framework for the relationship between the GEI and green development in China from the perspective of economic agglomeration and green patent application. Simultaneously, for regions with different levels of economic development and resource endowment in China, effective use of the positive impact of the GEI and promoting regional green development balance is of great significance. Microdata at the district and county level as well as enterprise data can help this study explore the relationship between GEI and green development more deeply. However, this study was not able to obtain relevant data because of its unavailability; therefore, further research is needed to explore the link between GEI and green development in more detail.

## Supporting information

S1 File (XLSX)
